# An updated review on immune checkpoint inhibitor-induced colitis: epidemiology, pathogenesis, treatment strategies, and the role of traditional Chinese medicine

**DOI:** 10.3389/fimmu.2025.1551445

**Published:** 2025-03-17

**Authors:** Huijing Dong, Yanmei Peng, Xinmeng Wang, Huijuan Cui

**Affiliations:** ^1^ China-Japan Friendship Clinical Medical College, Beijing University of Chinese Medicine, Beijing, China; ^2^ Department of Oncology, Fangshan Hospital Beijing University of Chinese Medicine, Beijing, China; ^3^ Department of Integrative Oncology, China-Japan Friendship Hospital, Beijing, China

**Keywords:** immune checkpoint inhibitors (ICIs), immune checkpoint inhibitor induced colitis (irColitis), traditional Chinese medicine (TCM), gut microbiota, review

## Abstract

Immune checkpoint inhibitor-induced colitis (irColitis) is a common and severe adverse reaction to immune checkpoint inhibitors (ICIs), significantly impacting the treatment outcomes and quality of life of cancer patients. Epidemiological studies indicate that the incidence of irColitis is associated with factors such as the type of ICIs, the patient’s gender, age, and medical history. Although the exact pathophysiology remains unclear, irColitis is thought to be related to immune system activation and dysregulation, gut microbiota imbalance, and impaired epithelial barrier function. This review summarized the epidemiology, clinical presentation, diagnostic criteria, and pathogenesis of irColitis. Additionally, the standard and novel therapeutic strategies of irColitis, including corticosteroids, biologics, and gut microbiota interventions, more importantly the potential and application of Traditional Chinese Medicine (TCM). Future researches call for deeper mechanistic investigations, the development of biomarkers, and reveal the integration of TCM therapies within individual immunotherapy frameworks.

## 1 Introduction

Immune checkpoint inhibitors (ICIs) have made significant advancements in cancer treatment, being widely used for non-small cell lung cancer (NSCLC), melanoma, gastric cancer, and other malignancies. This has transformed the treatment landscape for both solid tumors and hematological malignancies. The ICIs are associated with a range of immune-related adverse events (irAEs) that can affect multiple organs, including dermatological reactions, hepatitis, myocarditis, colitis, and others (1). Among these, immune checkpoint inhibitor-induced colitis (irColitis) is one of the most common and severe adverse effects. Patients with irColitis typically experience diarrhea, abdominal pain, and hematochezia, and even bowel perforation in severe cases. The irColitis significantly impacts patients’ quality of life, more seriously, causing drug dose reduction, discontinuation, or even death. The pathogenesis of irColitis remains unclear, but it is likely associated with immune system activation and dysregulation, gut microbiota imbalance, and impaired epithelial barrier function (2–4). Current treatments include corticosteroids and immunosuppressive agents; however, some patients exhibit inadequate responses or develop resistance, highlighting the urgent need for new therapeutic options for irColitis.

Under the principle of treatment based on syndrome differentiation, Traditional Chinese Medicine (TCM) has accumulated extensive experience in treating irAEs. For irColitis, TCM aims to restore the body’s balance and improve immune and intestinal functions (5). This holistic approach, along with syndrome differentiation and individualized treatment, have gradually demonstrated unique advantages.

This review aims to summarize the recent advances in the pathogenesis, clinical features, and TCM-based treatments of irColitis, while also exploring potential future research directions in this field. The goal is to provide new insights into clinical practice and offer reference points for future research endeavors.

## 2 Incidence and risk factors of irColitis

### 2.1 Incidence and risk factors of irColitis in previous literature

Studies have demonstrated that the incidence of irColitis varies significantly among different patient populations. A systematic review and meta-analysis (6) revealed that the incidence of colitis in patients treated with anti-programmed cell death protein 1 (PD-1) antibodies alone was 2%. In contrast, the use of Cytotoxic T-lymphocyte-associated antigen 4 (CTLA-4) inhibitors, such as ipilimumab, resulted in a notable increase in the incidence of colitis to 7%. In CheckMate 920 (7), the combination of nivolumab and ipilimumab led to an 18% incidence rate of all-grade colitis, with a 7% incidence rate of grade 3 or higher. As a consequence, one participant discontinued treatment. These findings suggested that the treatment modality of ICIs significantly influences the incidence of colitis. Other potential risk factors for irColitis have been identified in a cohort study of data from the Explorys, a US population database, indicating that irColitis occurred in 3.6% of patients. The study identified several risk factors associated with an increased likelihood of developing irColitis, including being female, white, older than 65 years, obese, and having a history of alcohol abuse (8).

Furthermore, a retrospective, comparative cohort study focused on the comorbid diseases associated with irColitis. This study showed patients with rheumatoid arthritis (RA) demonstrated a lower incidence of irColitis than those without RA, but the difference was not statistically significant(6 cases [7%] vs. 28 cases [14%]; p = 0.094) (9). This observed difference may be due to the distinct underlying pathological and immune mechanisms between irColitis and RA.

### 2.2 Incidence and risk factors of irColitis in clinical trials from the past five years

To characterize the epidemiology of irColitis, we reviewed clinical studies published on PubMed between 2020 and 2024 (10–49). The literature search used the keywords “Immune checkpoint inhibitor” and “colitis.” Clinical trials published in the past five years were selected based on the following inclusion criteria: (1) studies included more than 10 participants to ensure statistical robustness, minimized random variation, and reduced biases associated with small sample sizes or case reports; and (2) studies explicitly reported the incidence of irColitis to directly compare, minimizing consistency across different studies. These criteria were established to enhance the reliability of our findings and minimize data heterogeneity. The search results are presented in **Table 1**, including the year, country, sample size, drug type, and dosage (with treatment cycles and study endpoints detailed in the **Supplementary Table**), the incidence of irColitis (overall and grades 3–4), and the number of patients who discontinued treatment or died due to irColitis.

**Table 1:** Incidence of irColitis in recent five years.

Author (Year)	Country	Tumor Type	Drug and Dosage	Number of patients	Male, N (%)	Age, median (range)	ECOG PS:n (%)	Any grade, N (%)	grade 3-4, N (%)	Discontinuation,N (%)	Death, N (%)
Diaz (10) (2022)	Global	CRC^1^	pembrolizumab 200 mg q3w	153	71 (46.4)	63.0 (24–93)	0:75 (49.0)	10 (7.0)	5 (3)	2 (1)	–
Shitara (11) (2024)	Global	Gastric or Gastroesophagealadeno Carcinoma,	pembrolizumab 200 mg q3w	402	288 (72)	64 (56–70)	0:302 (75.0); 1:100 (25.0)	19 (5.0)	10 (3)	–	–
Lynch (12) (2023)	United States	Classic Hodgkin Lymphoma	pembrolizumab 200 mg q3w	30	12 (40)	33 (18-69)		1 (3.0)	0	–	–
Ready (13) (2023)	Global	NSCLC^2^	nivolumab (240 mg q2w) +ipilimumab (1 mg/kg q6w)	391	236 (60.4)	65.0 (26–89)	0-1	11 (2.8)	8 (2.0)	–	**-**
				139	90 (64.7)	67.0 (39–90)	2	3 (2.2)	1 (0.7)	–	–
Tykodi (14) (2022)	United States	RCC^3^	Nivolumab (3 mg/kg)+ ipilimumab (1 mg/kg)	52	36 (69.2)	64 (23–86)		7 (13.5)	4 (7.7)	–	–
Frentzas (15) (2024)	Australia	Solid Tumors	Ivonescimab (d1 and d15 of q28d) 0.3、1、3、10、20 and 30 mg/kg	52	18 (35.3)	63 (30, 76)	0:33 (64.7); 1:18 (35.3)	3 (5.9)	2 (3.9)	–	–
Schoenfeld (16) (2022)	United States	NSCLC	durvalumab 1,500 mg q4w/tremelimumab 75 mg q4w+ HFRT^5^	78	50 (64)	66 (59-72)	0:20 (26); 1:57 (73); 2:1 (1)	1 (3.8)	1 (3.8)	–	–
Oaknin (17) (2024)	Global	Cervical Cancer	nivolumab 240 mg q2w	19	0	51 (43–57)	0:10 (53); 1:8 (42)	0		–	–
			nivolumab 3 mg/kg q2w + ipilimumab 1 mg/kg q6w	45	0	48 (41–55)	0:23 (51) 1:22 (49)	1 (2)	1 (2)	–	–
			nivolumab 1 mg/kg q3w+ ipilimumab 3mg/kg q3w for 4cycles, followed by nivolumab 240 mg q2w	112	0	46 (38.5–54)	0:52 (46); 1:52 (46)	13 (12)	6 (5)	–	1 (0.9)
Monge (18) (2023)	United States	CRC	PexaVec+durvalumab 1500 mg q28d	16	6 (37)	53.5 (28-69)	0:11 (69); 1:5 (31)	0		–	–
			PexaVec+a single dose of tremelimumab (day1)+durvalumab 1500 mg q28d	18	8 (44)	56.5 (28-76)	0:16 (89); 1:2 (11)	2 (11)	1 (6)	1 (6)	–
Necchi (19) (2024)	Global	Bladder Cancer	pembrolizumab 200 mg q3w	132	104 (79)	72 (64.5–77.5)	0:101 (77); 1:28 (21); 2:3 (2)	3 (2)	3 (2)	1 (1)	–
Grimm (20) (2023)	Europe	RCC	nivolumab 240 mg q2w;nivolumab 3mg/kg+ipilimumab 1 mg/kg q3w	207	147 (71)	65 (57–71)		16 (8)	13 (6)	–	–
Lakhani (21) (2024)	Global	Solid Tumors	retifanlimab 3 mg/kg q2w	134	42 (31)	60 (18-86)	0:42 (31); 1:92 (69)	5 (4)	3 (2)	4 (3)	–
Schöffski (22) (2023)	Belgium,France,Denmark, United States	Soft-Tissue Sarcoma	Olaratumab (15or20 mg/kg, d1d8)+ pembrolizumab (200 mg,d1)	41	15 (36.6)	56.83 (43.76-69.9)	0:19 (46.3); 1:22 (53.7)	–	2 (4.9)	–	–
Zhao (23) (2024)	Global	NSCLC	SBRT (24 Gy in three fractions) with sequential tislelizumab (2 cycles of 200 mg) and chemotherapy	46	42 (91)	62 (58–65)	0:36 (78); 1:10 (22)	2 (4)	1 (2)	1 (2)	–
Saba (24) (2024)	United States	HNSCC^4^	nivolumab 240mg q2w and IMRT^6^ reirradiation	51	42 (82)	62 (56-67)	0:13 (25); 1:36 (71); 2:2 (3.9)	2 (3.9)	1 (2)	1 (2)	–
Emamekhoo (25) (2022)	United States	RCC	nivolumab 3mg/kg +ipilimumab 1mg/kg q3w; followed by nivolumab 480mg/4weeks	28	24 (86)	60 (38-87)		5 (18)	2 (7)	1 (4)	–
Morano (26) (2022)	Italy	CRC	temozolomide+nivolumab 480 q4w+ipilimumab 1 mg/kg q8w	33	17 (52)	58 (53-65)	0:22 (67); 1:11 (33)	6 (18)	1 (3)	–	–
Kim (27) (2024)	France	Squamous Cell Carcinoma of the anus	atezolizumab 800 mg, q2w+Chemotherapy	64	18 (28)	63.2 (56.0–71.9)	0:37 (58); 1:27 (42)	1 (2)		–	–
George (7) (2022)	United States	RCC	nivolumab 6 mg/kg+ipilimumab 1 mg/kg q8w; alternating with nivolumab 480 mg q8w, staggered q4w	106	86 (81.1)	64.5 (40–84)		15 (14.2)	8 (7.5)	4 (3.8)	–
Ferris (28) (2022)	United States	HNSCC	Cetuximab, Radiotherapy, and Ipilimumab (1,3,10mg/kg)	18	18 (95)	57 (43–74)	0:15 (79);1:4 (21)	1 (6)	1 (6)	1 (6)	–
Xiao (29) (2022)	United States	Solid Tumors	Pembrolizumab 200 mg q3w and SBRT^7^	73					2 (3)	–	–
Marabelle (30) (2020)	Global	Solid Tumors	pembrolizumab200mg q3w	233	96 (41.2)	60.0 (20-87)	0:113 (48.5) 1:120 (51.5)	9 (3.9)	2 (0.9)	–	–
Stratigos (31) (2021)	Canada, Europe, and the USA.	advanced Basal Cell Carcinoma	cemiplimab 350 mg q3w	84			0 or 1		4 (5)	–	–
Qian (32) (2021)	United States	Melanoma	ipilimumab, nivolumab, or pembrolizumab, or a combination of these	299	197 (66)			54 (18)		–	–
Tawbi (33) (2021)	United States	Melanoma brain metastases	nivolumab 1 mg/kg +ipilimumab 3 mg/kg q3w; nivolumab 3 mg/kg	Asymptomatic patients101	68 (67)	59 (51–66)	0 or 1	7 (7)	7 (7)	–	–
Ascierto (34) (2020)	Global	Melanoma	ipilimumab 10 mg/kg q3w	453					2 (0.4)		1 (0.2)
Goldberg (35) (2020)	United States	NSCLC with brain metastasis	pembrolizumab 10mg/kg q2w	42	14 (33)	60 (56-71)	0:4 (10);1:38 (90)	5 (11.9)	1 (2)	–	–
Campbell (36) (2021)	United States	RCC	tremelimumab 10mg/kg q4w	29	24 (83)	59 (23-82)	0:18 (62);1: 9 (31);2: 2 (7)	6 (21)	6 (21)		
Cacciotti (37) (2020)	United States	Central Nervous System Tumors	ipilimumab,nivolumab and/or pembrolizumab	11	6 (55)	13.9 (4.1-20.7)	–	3 (27.3)	1 (9)	2 (18.2)	–
Gao (38) (2020)	United States	high-risk Urothelial Carcinoma	durvalumab (1500mg) + tremelimumab (75 mg)	28	20 (71)	71 (24–83)	–	–	2 (7)	–	–
Brastianos (39) (2021)	United States	Leptomeningeal Carcinomatosis	nivolumab+ipilimumab, dosage vary depending on the type of cancer.	18	7 (38.9)	54 (36–70)	0:4 (22.2);1: 10 (55.6);2:4 (22.2)	1 (5.6)	–	1 (5.6)	–
Kawazoe (40) (2020)	Japan	Gastric/Gastroesophageal Junction Cancer	Pembrolizumab 200 mg q3w	54	43 (79.6)	66 (32–75)	0:46 (85.2);1:8 (14.8)	5 (9.3)	3 (5.6)	–	–
Tolaney (41) (2020)	United States	Breast Cancer	pembrolizumab, 200 mg, q3w	44	0	58 (30-76)	0:35 (80);1:9 (21)	2 (5)	2 (5)	–	2 (5)
Sanborn (42) (2021)	Global	Solid Tumors	pacmilimab (0.3, 1, 3, or 10 mg/kg) +ipilimumab (3 or 6 mg/kg) q3w for 4 doses, followed by pacmilimab monotherapyq2w.	27	11 (41)	56 (28–70)	0:11 (41);1:16 (59)	2 (7)	2 (7)	1 (3.7)	–
Desai (43) (2020)	Australia, Korea, New Zealand and Taiwan	Solid Tumors	tislelizumab	451	246 (54.5)	61.0 (18.0- 81.0)	0:169 (37.5);1:282 (62.5)	6 (1.3)	3 (0.7)	–	–
Diefenbach (44) (2020)	United States	Hodgkin Lymphoma	brentuximab1-8mg/kg, nivolumab3mg/kg, and ipilimumab1mg/kg	22	11 (50)	35 (19–60)	0:12 (55);1-2:10 (45)	1 (2)	1 (2)	–	–
Apolo (45) (2020)	United States	Genitourinary Malignances	cabozantinib 40 mg/d, nivolumab 3 mg/kg, and ipilimumab 1 mg/kg	24				2 (8)	2 (8)		
Yap (46) (2021)	Global	Mesothelioma	pembrolizumab 200 mg q3w	118					3 (2.5)		
McDermott (47) (2021)	Global	CRC	pembrolizumab 200 mg q3w	110	86 (78.2)	64 (29-87)	–	6 (5.5)	6 (5.5)	–	–
Boutros (48) (2020)	France	Melanoma	ipilimumab10 mg/kg q3w and radiotherapy	19	10 (53)	58 (35–85)	0 or 1	6 (32)	2 (10.5)	–	–
Felip (49) (2020)	Global	NSCLC	nivolumab 3mg/kg q2w	811	640 (78.9)	66 (31–86)	0:173 (21.3);1:534 (65.8);2:103 (12.7);3:1 (0.1)	7 (0.9)	5 (0.6)	2 (0.2)	–

^1^ CRC, colorectal cancer; ^2^ NSCLC, non-small cell lung cancer; ^3^ RCC, renal cell carcinom; ^4^ HNSCC, head and neck squamous cell carcinoma; ^5^ HFRT, Hypofractionated Radiotherapy; ^6^ IMRT, Intensity-Modulated Radiation Therapy; ^7^ SBRT, Stereotactic Body Radiation Therapy.

As shown in **Table 1**, these studies focused on the incidence of irColitis across various malignancies. Among the 41 included studies, 39 were prospective, including 28 phase I/II trials and 7 phase III/IV trials. These trials investigated various types of cancer, including melanoma, NSCLC, Renal Cell Carcinoma (RCC), Colorectal Cancer (CRC), Head and Neck Squamous Cell Carcinoma (HNSCC), gastroesophageal adenocarcinoma, classical Hodgkin lymphoma, cervical cancer, bladder cancer, soft tissue sarcoma, and anal cancer. The incidence of colitis varied significantly, ranging from 0 to 32%, with the rate of grade 3 or higher events varying between 0 and 21%.

These differences were primarily influenced by tumor type, the immunotherapy drugs and doses used, treatment frequency, and patient characteristics. Regarding the relationship between immunotherapy drug types and the incidence of irColitis, our findings aligned with previous literature. Compared to treatment with a single drug, the incidence of irColitis was significantly higher in combination therapies. For example, the incidence of irColitis reached 18% in patients treated with nivolumab and ipilimumab (3 mg/kg + 1 mg/kg) (25). In contrast, monotherapy with nivolumab at 3 mg/kg showed a lower incidence of 0.9% compared to ICIs combination therapy (49). Additionally, high-dose and high-frequency application of ICIs appeared to be associated with an increased incidence of irColitis. For instance, pembrolizumab at 10 mg/kg every 2 weeks resulted in an overall irColitis incidence of 11.9% (35), whereas fixed-dose pembrolizumab at 200 mg every 3 weeks led to an overall irColitis incidence of 2%-9.3% (19, 40). Furthermore, patients with different tumor types had varying prevalences of irColitis. For example, in CRC, the incidence of irColitis with pembrolizumab was 5.5%-7% (10, 47), whereas a much lower incidence of 2% in bladder cancer (19). Age was also found to be related to the occurrence of irColitis. In patients with a median age of 13.9 years old, the incidence of irColitis was 27.3% (37), a higher incidence than that in elderly patients (aged over 55 years), ranging from 0.9% to 32% (48, 49). In contrast, the incidence was lower in middle-aged population (30-55 years), ranging from 0% to 12% (12, 17, 18, 44). However, Necchi found age may not be the primary factor compared to other factors (19); in this study, the median age of patients was 72 years old, but the incidence of irColitis reported 2%.

As shown in **Table 1**, 13 of 41 studies reported discontinuation of immunotherapy due to irColitis (7, 10, 18, 19, 21, 23–25, 28, 37, 39, 42, 49). The ICIs used in these studies included single-agent pembrolizumab, tremelimumab, durvalumab, retifanlimab, tislelizumab, nivolumab, and ipilimumab, as well as combinations of nivolumab with ipilimumab, and pacmilimab with ipilimumab. Cacciotti’s study observed the highest discontinuation rate at 18.2% (37); this study focused on children and young adults with recurrent pediatric high-grade central nervous system (CNS) tumors, which were treated with ipilimumab, nivolumab, and/or pembrolizumab. The high discontinuation rate may be attributed to the combined effects of tumor malignancy, patient age, and the use of combination immunotherapy. Additionally, two studies reported a 6% discontinuation rate due to irColitis (18, 28). Three studies reported irColitis-related deaths. One case involved a female patient with metastatic cervical cancer, who received nivolumab (1 mg/kg) and ipilimumab (3 mg/kg) every 3 weeks for 4 cycles, followed by nivolumab (240 mg) every 2 weeks (17). Another case involved a patient with advanced melanoma treated with ipilimumab (34). Additionally, two cases involved females with receptor-positive, ERBB2-negative metastatic breast cancer, who died of eribulin and pembrolizumab-induced irColitis due to sepsis (41).

However, we acknowledge the potential for selection bias in our review. Focusing on published clinical trials, we have inadvertently excluded smaller retrospective studies and case reports, potentially underestimating rare presentations of irColitis. Additionally, publication bias may have influenced the reported incidence rates. To mitigate these limitations, we conducted a comprehensive keyword search and systematically reviewed the reference lists of relevant articles.

## 3 Clinical features, diagnosis, and grading of irColitis

### 3.1 Clinical features

IrColiti typically manifests as diarrhea, the primary clinical symptom. Diarrhea has been found to occur early during immunotherapy. The median onset of diarrhea/colitis typically occurs around 3 to 6 months after treatment with anti-PD-1/Programmed cell death-ligand 1 (PD-L1) agents, while earlier for anti-CTLA-4 agents at around 6 to 8 weeks (50, 51). However, there are also cases of relapse occurring 1-2 years after treatment discontinuation (52). Patients often experience frequent bowel movements, with stools containing mucus or blood. Symptom severity ranges from mild to severe and may significantly impact the patient’s quality of life. Besides diarrhea, patients with irColitis frequently report other gastrointestinal symptoms, including abdominal pain, fever, and, in some cases, upper gastrointestinal symptoms such as dyspepsia, reflux, or heartburn (53, 54).

In addition to the typical symptoms of diarrhea and abdominal pain, irColitis may present with atypical manifestations, including anorexia, significant weight loss or dehydration due to prolonged diarrhea, and fatigue. These systemic symptoms further complicate the clinical management of irColitis (55).

These clinical manifestations may resemble those of other gastrointestinal disorders, making it essential to differentiate irColitis from infections, inflammatory bowel disease (IBD), or ischemic colitis. A careful differential diagnosis is required for proper treatment strategies.

### 3.2 Endoscopic and pathological findings

The pathological changes associated with irColitis are diverse and complex. Although the condition is typically characterized by pancolitis, irColitis may occasionally affect only the descending colon (56, 57). These changes not only influence clinical diagnosis but also have significant implications for treatment strategies and patient prognosis. Common endoscopic findings included erythema, loss of vascular pattern, granular appearance, and ulcerations; however, normal mucosa might also be observed in some cases (57, 58). Therefore, a biopsy has been recommended for suspect irColitis patients.

According to existing literature, the pathological changes associated with irColitis could be classified into several types: (1) Active colitis is the most common histopathological pattern of irColitis. This form is characterized by neutrophil infiltration in the lamina propria, cryptitis, and crypt abscesses. Crypt atrophy or loss was uncommon but could be found Increased epithelial apoptosis and intraepithelial lymphocytes could also be observed (57); (2) Chronic active colitis is commonly observed after long-term ICI treatment. Histopathological findings include persistent lymphocytic infiltration and disruption of intestinal architecture, such as crypt distortion and pseudopyloric or Paneth cell metaplasia (57). Crypt abscesses and ulceration of the intestinal epithelium may also be present (59). (3) Microscopic colitis often appears normal on endoscopy, making it difficult to detect. Histopathological changes do not show significant acute or active inflammation. However, histological examination may reveal increased intraepithelial lymphocytes and lymphoplasmacytic infiltration in the lamina propria (57, 60). Microscopic colitis is potentially required for additional treatment with oral or intravenous corticosteroids and/or nonsteroidal immunosuppressive agents. In addition to these three common types, other patterns, such as increased apoptosis (61), ischemic colitis (58), and nonspecific inflammatory reactive changes (57), have also been observed.

### 3.3 Diagnosis and grading of irColitis

The diagnosis of irColitis primarily depends on clinical manifestations, laboratory tests, imaging studies, and endoscopic evaluation, making it a complex process that integrates various standards and methodologies. Currently, several grading systems are employed to assess clinical symptoms, including the Common Terminology Criteria for Adverse Events (CTCAE) (62), the treatment guidelines for irAEs from the European Society for Medical Oncology (ESMO) (63), the American Society of Clinical Oncology (ASCO) (64), and Chinese Society of Clinical Oncology (CSCO) Guidelines for the management of toxicities related to ICIs (65) (**Table 2**). However, the ESMO/ASCO/CSCO guidelines do not address irColitis separately; instead, they group irColitis and diarrhea together for grading and management.

**Table 2:** Grading systems for irColitis.

Guideline	Grade			
	I	II	III	IV
CTCAE5.0^a^ (62)	Asymptomatic; clinical or diagnostic observations only; intervention not indicated	Abdominal pain; mucus or blood in stool	Severe abdominal pain; peritoneal signs	Life-threatening consequences; urgent intervention indicated
ESMO (63)	increase of <4 stools/day over baseline	increase of 4-6 stools/day over baseline	increase of≥7 stools/day	Life-threatening consequences or any grade of diarrhea and one of the following: hematochezia, abdominal pain, mucus in stool, dehydration, fever
ASCO (64)	Increase of < 4 stools/day over baseline; mild increase in ostomy output compared with baseline	Increase of 4-6 stools/day over baseline; moderate increase in ostomy output compared with baseline	Increase of ≥7 stools/day over baseline; incontinence; hospitalization indicated; severe increase in ostomy output compared with baseline, and limiting self-care ADL	Life-threatening consequences; urgent intervention indicated
CSCO (65)	Asymptomatic; requires only clinical or diagnostic observation (diarrhea ≤ 4 times/day).	Abdominal pain; fecal mucus or blood (diarrhea frequency 4-6 times/day).	Severe abdominal pain; changes in bowel habits; requires pharmacological intervention; signs of peritoneal irritation (diarrhea frequency ≥ 7 times/day).	Life-threatening symptoms; requires urgent intervention.

^a:^A disorder characterized by inflammation of the colon.

Endoscopic examination plays a crucial role in diagnosing irColitis as the golden standard (66, 67). However, due to the similar symptoms between irColitis and other gastrointestinal disorders, additional diagnostic tests are essential for differentiating diagnosis. For example, laboratory blood tests and stool analyses can help differentiate irColitis from infections and IBD. The imaging modalities of CT and MRI are more valuable in evaluating the extent and severity of intestinal inflammation, as well as in managing complex cases of irColitis that may pose risks for endoscopic procedures (66).

Beyond conventional diagnostic methods, recent advancements in irColitis biomarkers have shown promise for predicting and diagnosing its occurrence. Anti-integrin αvβ6, a heterodimeric cell adhesion receptor, has been identified as a potential biomarker. A study revealed that Anti-integrin αvβ6 autoantibodies were significantly more prevalent in irColitis patients than in control groups, including patients with other organ irAEs, cancer patients without irAEs, and healthy volunteers (30.8% vs. 1.9%) (68). Furthermore, the presence of Anti-integrin αvβ6 autoantibodies was associated with disease activity, supported by characteristic endoscopic findings, high-grade irColitis, and steroid resistance (68). A single-cell sequencing study of 13 irColitis patients revealed that increased mucosal Regulatory T cells (Tregs), ITGAE^Hi^ CD8 tissue-resident memory T cells (TRMs) expressing C-X-C motif chemokine ligand 13 (CXCL13) and T helper 17 (Th17) gene programs, and recirculating ITGB2^Hi^ CD8 T cells may serve as potential biomarkers for irColitis (69). Another study involving 15 irColitis patients showed that activated CD8+ TRMs cells express high levels of transcripts for checkpoint inhibitors and interferon-gamma and identified Interferon-Gamma-Producing CD8+ TRMs as potential targets (3).

## 4 The possible mechanism of irColitis

**Figure 1:**
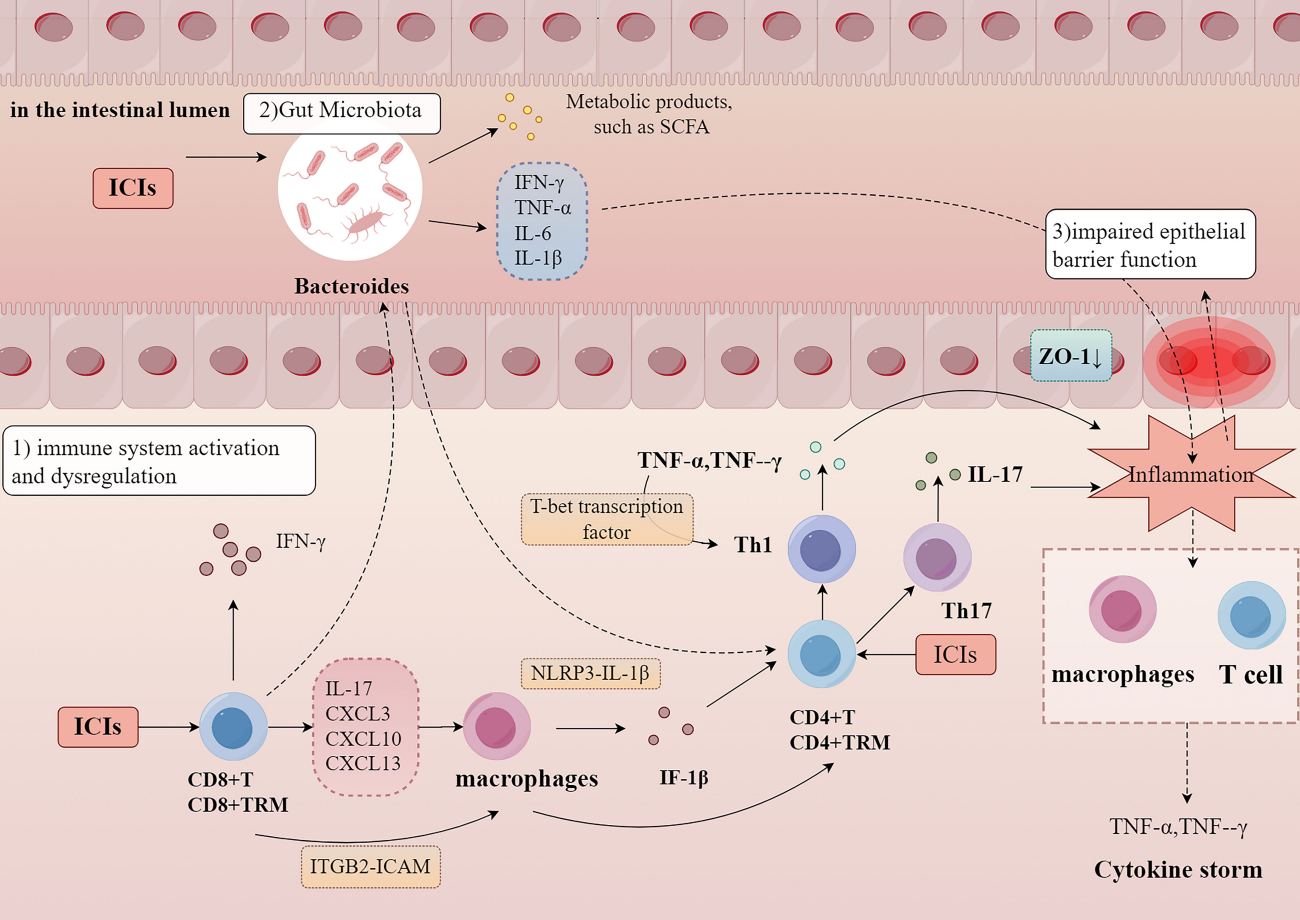
The possible mechanism of irColitis. ICIs induce immune system activation and dysregulation, resulting in excessive T cell and macrophage activation, along with increased pro-inflammatory cytokine release. Gut microbiota, particularly *Bacteroides*, contribute to inflammation by producing metabolic byproducts and modulating cytokine expression. Additionally, epithelial barrier dysfunction, characterized by reduced ZO-1 expression, further amplifies inflammation, ultimately leading to immune dysregulation and a cytokine storm. (TRM, tissue-resident memory T cells; SCFA, Short-Chain Fatty Acids; ZO-1, Zonula Occludens-1; IFN, Interferon; TH, T helper cells; IL, Interleukin; TNF, Tumor Necrosis Factor. Detailed abbreviations are presented in **Supplementary Table 2**. Image created with Figdraw.com).

Recent advances in research have illuminated key pathological processes, such as T cell overactivation, pro-inflammatory cytokine production, gut microbiota dysbiosis, and epithelial barrier dysfunction, providing novel insights into irColitis pathogenesis (**Table 3**, **Figure 1**).

**Table 3:** The possible mechanism of irColitis.

Author	Year	Experimental subject	Mechanism of Effect/effect cells	Effect
Tomm (4)	2024	Biopsies of patients with irColitis	–	Pathology features: intraepithelial lymphocytes↑, epithelial cell apoptosis↑, and active inflammation
Malik (70)	2023	mouse model of acute colitis	upregulation of IFNγR-STAT-IRF1 pathway	IFN-γ↑, IRF1↑, STAT activation; GM-CSF+CD4+ T cells↑, IL-17↑
Lo (71)	2024	irColitis mouse model with dissimilar gut microbiota composition	–	CD4+ T cells↑, IFN-γ↑, Th1 response↑, Treg↑; WildR microbiota (+)
Zeng (72)	2024	colitis mouse model	activation of JAK2/STAT3/SOCS3 pathway	TIPE2↑, tight junction proteins (Occludin, Claudin-1, ZO-1)↓, SOCS3↑ Epithelial barrier dysfunction
Reschke (73)	2022	patients with irColitis	–	IFN-γ↑, CXCL9↑, CXCL10↑, CXCL11↑, IL-17↑; Th1 response↑; CD8 + TRM↑, CD4 + TRM↑
Halsey (74)	2023	patients with irColitis	–	Collinsella↓, Bifidobacterium↓, Tyzerella↑
Thomas (69)	2024	colon mucosa cells from patients with irColitis	–	IL17A↑, IL26↑, CXCL13↑, Th17↑; CD8 + Tcell↑, CD8 + TRM↑, CD4 + TRM↑; intravascular effector memory populations↑; Epithelial defects: stem cells↓, transit amplifying cells↑, and top crypt epithelial cells (with ISGs, CASP1, ZBP1, ICAM1, CD274/PD-L1, CXCL10/11)↑, aquaporin (AQP) water channel genes↓
He (75)	2024	colon biopsies and blood from patients with irColitis	–	CD8 + T↑, CD8 + TRM↑, CD4 + TRM↑; perturbed stromal metabolism
Ghosh (76)	2024	patients with irColitis	–	CD8 + TRM↑, CD4 + TRM↑,IL-17↑; CXCL8↑, GM-CSF↑, CXCL1↑, IL-6↑, TNF-α↑
Ye (77)	2024	irColitis mouse model	DICB (Double immune checkpoint blockade)/ TNF-α/gut microbiota loop	TNF-α↑, IL-6 ↑, IL-1β↑; Bacteroides↑

"↑" means an increase, while "↓" represents a decrease in expression.

### 4.1 The activation and disorder of immune system

#### 4.1.1 Excessive T cell activation and pro-inflammatory cytokine production

By blocking inhibitory PD-1/CTLA-4 pathways, ICIs activate inhibited T cells thereby enhancing the immune system’s ability to attack tumor cells; however, this activation can cause T cell overactivation, resulting in the loss of immune tolerance, particularly in mucosal tissues, which is a key driver of irColitis (75).

Substantial evidence supports that CD8+ T cells are the key effector cells in irColitis (2). A cross-sectional study of clinical and pathological data analyses from patients with anti-CTLA-4/PD-1 colitis demonstrated that CD8+TRMs constituted the majority of activated TRMs in irColitis, with the degree of activation correlating with clinical and endoscopic severity. Notably, these CD8+ TRMs exhibited significantly higher expression of Interferon-gamma (IFN-γ), which further promoted TRMs activation. This finding was complemented by evidence of upregulation of classic Janus kinase/Signal transducer and activator of transcription (JAK/STAT) components in the IFN-γ signaling pathway in irColitis (3). Collectively, the upregulation of IFN-γ signaling represents a key pathological feature of irColitis, with the JAK/STAT signaling pathway playing a central role in this process, thereby providing a theoretical basis for potential therapeutic strategies, such as JAK inhibitors (74).

Thomas et al. applied single-cell multi-omics to analyze approximately 300,000 cells from the colonic mucosa and blood samples of patients with irColitis. Their findings revealed that the expansion of mucosal Tregs, CD8+ TRMs, and recirculating CD8+ T cells are hallmark features of irColitis, particularly T cells expressing pro-inflammatory gene programs, such as CXCL13 and Th17. In patients with irColitis, 1.9% of CD8+ T cells expressed Interleukin-17 (IL-17) A, while IFN-γ and CXCL13 were significantly upregulated in intestinal tissue samples (69). These cytokines synergistically impair epithelial integrity and drive chronic inflammation, further elucidating the mechanisms underlying irColitis. CD4+ T cells can differentiate into various phenotypes, including T helper 1 (Th1), Th17, and Treg cells. Th1 cells primarily secrete IFN-γ, while Th17 cells produce cytokines such as IL-17, which is closely associated with the pathogenesis of intestinal inflammation. Numerous studies have indicated that the overactivation of CD4+ T cells contributes to the development and progression of irColitis (78). To further elucidate the roles of CD4+ T cells in irColitis, Lo et al. established an irColitis mouse model using anti-CTLA-4 treatment. Their findings demonstrated that CTLA-4 blockade significantly promotes the accumulation of IFN-γ+ CD4+ T cells, resulting in intestinal tissue damage, which is considered the initial trigger of irColitis (71). Additionally, they observed that IFN-γ expression was closely linked to the activation of the T-bet transcription factor, which amplified the Th1 response and underscores the Th1-biased inflammatory response in irColitis. Furthermore, a recent study analyzing colon biopsies and blood samples from irColitis patients revealed that CD4+ TRMs were progenitors of cytotoxic effectors such as CD8+ T cells, thereby identifying CD4+ TRMs as novel therapeutic targets (75).

#### 4.1.2 Immune cell interactions

Interactions among immune cells play a pivotal role in disease pathogenesis when irColitis occur. ICIs relieve immune suppression, thereby altering the crosstalk among various immune cell populations within the intestines. These interactions notably include those between T cells and macrophages, dendritic cells, and epithelial cells.

Recent research has shown that activated CD8+ T cells interact with macrophages through C-X-C Chemokine Receptor Type 3 (CXCR3) and macrophage-derived C-X-C Motif Chemokine Ligand 10 (CXCL10), as well as Integrin beta 2-Intercellular Adhesion Molecule (ITGB2-ICAM) pathways. These interactions facilitated macrophage recruitment and activation, initiating localized inflammatory responses in the intestine (55, 69). To further investigate this mechanism, Ankit et al. created an immunodeficient mouse model. Their results demonstrated that conditional deletion of Major Histocompatibility Complex class II (MHC-II) in macrophages reduced the proportion of Granulocyte-macrophage colony-stimulating factor (GM-CSF) + CD4+ T cells by 70%, from 18.3% to 5.5%. This suggests that macrophages present antigens via MHC-II and release Interleukin-1 beta (IL-1β), which promotes the differentiation of CD4+ T cells into GM-CSF+ subsets, contributing to irColitis. Moreover, the study revealed that inhibiting the Nucleotide-binding oligomerization domain (NOD)-like receptor family pyrin domain containing 3 (NLRP3) inflammasome significantly reduced IL-1β production in macrophages, which in turn decreased the population of GM-CSF+ CD4+ T cells (70). These findings underscored the role of macrophages in regulating T cell activation through the NLRP3-IL-1β axis, offering potential therapeutic targets for irColitis.

The interaction between T cells and epithelial cells plays a crucial role in the pathogenesis of irColitis. Epithelial cells enhance the pro-inflammatory activity of CD4+ T cells via MHC-II-mediated antigen presentation. Moreover, the absence of IFN-γ signaling in epithelial cells exacerbates the pathological activation of CD4+ T cells (70). Elevated expression of PD-L1 in intestinal epithelial cells has also been observed; however, this pathway becomes dysregulated in the presence of ICIs, leading to uncontrolled T cell cytotoxic responses. This dysregulation indicates a loss of immune tolerance in patients with irColitis (79).

### 4.2 The dysbiosis of gut microbiome

Recent metabolomics evidence has shown that dysbiosis-induced alterations in lipid and amino acid metabolism contribute to gut inflammation in irColitis.

Ye et al. suggested that certain microbial species, particularly within the Bacteroides genus, exacerbated irColitis through pro-inflammatory effects. Positive correlations between bacteroides and inflammatory factors, such as tumor necrosis factor alpha (TNF-α), interleukin (IL)-6, and IL-1β, highlighted the interactions between microbiota, metabolites, and inflammation. Additionally, gut microbiota dysbiosis intensified TNF-α signaling, further disrupting microbial composition and metabolite production, thereby aggravating irColitis (77).

Activated CD8+ TRMs may contribute to the upregulation of IFN-γ signaling and the development of irColitis by mediating immune responses to both commensal or pathogenic microbes. This suggests that alterations in the microbiome play a critical role in the pathogenesis of irColitis. Therapeutic strategies targeting the gut microbiome, such as fecal microbiota transplantation (FMT), may provide a promising approach to mitigate this condition (3).

The microbiome plays a critical role in the activation of CD4+ T cells and the onset of colitis. Severe intestinal inflammation induced by anti-CTLA-4 treatment occurs only in mice with a fully functional, free-living microbiome. This suggests that the composition of the gut microbiota not only influences CD4+ T cell activation but may also exacerbate inflammatory responses through interactions with the immune system (71).

In conclusion, we find that gut microbiota dysbiosis both drives and results from irColitis. Dysbiosis amplifies local inflammation through pro-inflammatory species and disrupted metabolic pathways, establishing a vicious cycle.

### 4.3 The disruption intestinal barrier

#### 4.3.1 Epithelial barrier dysfunction

The intestinal epithelial barrier is essential for preventing antigen transfer, and its damage is considered one of the key pathological features of irColitis.

As discussed earlier, the enrichment of pro-inflammatory cytokines, such as IFN-γ and TNF-α, is observed in irColitis. Previous evidence supported that these cytokines could increase epithelial cell apoptosis, thereby impairing the intestinal epithelial barrier and contributing to bowel inflammation, a key factor in the development of irColitis (72). Epithelial defects, including upregulation of apoptosis-related genes (Caspase-1 [CASP1], Caspase-8 [CASP8]), interferon-stimulated genes (STAT1, PD-L1), and pro-inflammatory marker genes (CXCL10), as well as downregulation of aquaporin water channel genes, suggest impaired water and solute transport in the intestines (69). Disruption of tight junction proteins, such as myosin light chain kinase (MLCK) and zonula occludens-1 (ZO-1), increased intestinal permeability and epithelial barrier dysfunction, allowing microbial products to activate immune cells, including T cells and macrophages. This triggered a cytokine storm involving TNF-α and IFN-γ, and further amplified inflammation in irColitis (80).

Interventions targeting the epithelial barrier, such as MLCK inhibitors, show promise in reducing inflammation while preserving the efficacy of tumor immunotherapy. This strategy may effectively manage irColitis by restoring barrier integrity.

#### 4.3.2 Alterations of intestinal structure

Epithelial remodeling, a key feature of irColitis, also represents the precursor to intestinal reconstruction. A clinical study highlighted significant structural alterations in the intestine during irColitis, including epithelial barrier disruption, crypt architectural distortion, and increased immune cell infiltration (4). These changes compromise intestinal integrity, allowing microbial antigens to reach immune cells, which perpetuates inflammation. Chronic inflammation is linked to abnormal epithelial regeneration and the loss of goblet cells, further impairing mucosal defense.

At the cellular and molecular level, evidence suggested that interferon signaling induces changes in epithelial differentiation, leading to a decrease in LGR5^Hi^ stem cells and an increase in transit-amplifying cells, along with the loss of mature epithelial cells and enrichment of interferon-induced epithelial subsets. Additionally, some cases of irColitis feature vascular and fibroblast remodeling, potentially driven by the expansion of angiogenesis-related genes (Hypoxia-inducible factor 1 alpha [HIF1A], Vascular Endothelial Growth Factor A [VEGFA]), highlighting the potential role of neovascularization in the pathological process of irColitis (69, 81).

## 5 Treatment of irColitis

The recommended treatment of irColitis primarily included corticosteroids, biologic agents, and certain non-pharmacological interventions. Moreover, TCM, as a natural therapeutic approach, has shown a promising potential in the treatment of this condition.

### 5.1 Corticosteroids

Corticosteroids are the first-line treatment for irColitis, primarily used to suppress T cell activation, thereby inhibiting the production of pro-inflammatory cytokines and regulating overactive innate and adaptive immune responses (82). The NCCN guidelines recommend that patients experiencing grade 2 or higher irColitis should discontinue immunotherapy and receive prednisone or methylprednisolone (1–2 mg/kg/day) until symptoms improve to grade 1 or lower. Corticosteroid tapering should then be performed over 4 to 6 weeks (83). However, some patients may develop resistance to corticosteroids or exhibit poor responses, with relapses occurring during dose reduction, complicating further management (82, 84). A retrospective study reported a clinical response rate of 70% (14/20) to corticosteroid treatment. Among these 14 patients, 2 (14%) experienced relapse during corticosteroid tapering and became steroid-dependent (85). Another retrospective analysis of 49 irColitis patients found that all received glucocorticoids as first-line treatment; however, immunotherapy was discontinued in 21 cases (86). In such cases, it is essential to promptly assess the patient’s clinical condition, differentiate irColitis from other gastrointestinal disorders, and identify potential complications. Alternative therapeutic options should then be considered based on this evaluation.

### 5.2 Biologic agents

For patients with corticosteroid resistance or inadequate response, biologic agents are an important therapeutic option. Recent studies have shown that anti-TNF-α monoclonal antibody infliximab and α4β7 integrin immunoglobulin G1 (IgG1) monoclonal antibody vedolizumab exhibited promising efficacy in the treatment of irColitis (82, 83). If no improvement is observed within 2 to 3 days after initiating corticosteroid therapy, infliximab or vedolizumab may be considered within 2 weeks of diarrhea onset while continuing corticosteroid treatment (83). Additionally, patients who relapse during corticosteroid tapering or after completing the corticosteroid regimen may also require additional immunosuppressive therapy (67). Infliximab, a second-line treatment, is widely used in corticosteroid-resistant patients. Clinical data demonstrate its effectiveness in severe acute colitis cases, with a retrospective study reporting a 71.4% remission rate for corticosteroid-resistant irColitis (87). Furthermore, infliximab has been shown to help maintain overall survival (OS) in these patients (88). A study comparing infliximab with corticosteroids demonstrated that infliximab resulted in a shorter median time to diarrhea resolution and corticosteroid tapering compared to the corticosteroid group without negatively affecting OS or time to treatment failure (TTF) (89). Another biologic agent, vedolizumab, is commonly administered intravenously at a dose of 300 mg (90). Although vedolizumab had not been as extensively studied in clinical applications as infliximab; a multicenter retrospective study demonstrated that vedolizumab treatment for corticosteroid-resistant irColitis resulted in sustained clinical remission in 84% of patients (90).

### 5.3 Gut microbiota

The gut microbiota plays a crucial role in regulating intestinal immune function. One study found that *Faecalibacterium prausnitzii* helped reduce intestinal toxicity and boost tumor immunity, enhancing the effectiveness of dual CTLA-4 and PD-1 checkpoint blockade (91). This suggests that supplementary probiotics could potentially reduce the risk of colitis while improving the response to immunotherapy. FMT is an emerging therapeutic approach that has been applied to patients with refractory irColitis. A study involving 12 patients with severe irColitis showed that FMT effectively improved clinical symptoms, with 92% of patients achieving clinical remission after treatment (74). This effect may be mediated through FMT’s influence on gut microbiota diversity and composition. Additionally, a significant reduction in total lymphocytes and CD8+ T cells was observed in complete responders, indicating that FMT mediates inflammation reduction in these patients (74).

### 5.4 Traditional Chinese medicine and irColitis

TCM has a long and rich history in treating digestive disease, with extensive experience in treating irAEs. It also offers valuable insights into the treatment of irColitis. TCM views the human body as an integrated whole. When the balance of Yin and Yang and the normal function of the internal organs are maintained, as described in the *Huangdi Neijing* (Yellow Emperor’s Inner Canon), “When Yin is in balance and Yang is hidden, the spirit is healthy,” and “When the vital Qi is preserved within, pathogens cannot invade.” Immunotherapy with ICIs can disrupt this balance while targeting tumors, leading to irAEs. In TCM, irColitis is often classified under categories such as “diarrhea (Xie Xie)” and “intestinal dysentery (Chang Pi).” The occurrence of irColitis is believed to be closely related to pathogenic heat, damp-heat, damage to the intestinal collateral, and spleen-stomach weakness. As the disease progresses, patients may also develop symptoms of Spleen and Kidney Yang deficiency (5). The treatment principles in TCM involve clearing heat and detoxifying, strengthening the spleen and resolving dampness, and warming and tonifying the spleen and kidneys to regulate immune function, balance the internal organs, alleviate symptoms, and promote intestinal recovery. Additionally, TCM offers various methods for treating “diarrhea,” including oral administration, moxibustion, acupoint application, and enemas (5). The rich experience in treating “diarrhea” in TCM can provide valuable insights for future experimental studies and clinical trials on natural product therapies for irColitis.

Since no specific studies have investigated the use of TCM for irColitis, we could retrieved related studies based TCM theory of “treating different diseases with same method”. In TCM, ulcerative colitis, Crohn’s disease, and irColitis share similar symptoms, including diarrhea, abdominal pain, and rectal bleeding. These conditions are classified under the categories of “diarrhea” or “intestinal dysentery”. Therefore, this study conducted a comprehensive review of clinical articles on TCM treatments for diarrhea-related diseases from databases of PubMed and CNKI. This review provides valuable insights for future research (**Table 4**). The PubMed search utilized the following keywords and MeSH terms: (“Traditional Chinese Medicine” OR “Chinese herbal medicine” OR “Chinese medicine”) AND (“ulcerative colitis” OR “Crohn’s disease” OR “ colitis “), focusing on clinical studies published within the past five years. A similar search strategy was applied to Chinese databases. The inclusion criteria were as follows: (1) studies reported clinical outcomes of TCM in the treatment of irColitis or IBD; (2) studies had a standard research design. Studies were excluded if they lacked specific outcome measures, had an unclear study design, or involved insufficient sample sizes that could compromise statistical validity. To minimize selection bias, representative studies from both English and Chinese databases were included, with a preference for those demonstrating high methodological rigor. However, potential selection bias remains due to publication bias and variations in study design, which is recognized as a limitation.

**Table 4:** The potential clinical experience of TCM in the treatment of irColitis.

Author (Year)	Number of patients	Treatment Method	Composition of treatment	Therapeutic Principles	Frequency	Therapeutic Effects	Type	TCM Diagnosis	Disease	Potential Mechanisms of Action
Yang (2020) (92)	70	Oral	*Gegen Qinlian decoction*: *Radix Puerariae* (Ge Gen), *Scutellariae Radix* (Huang Qin), *Coptidis Rhizoma* (Huang Lian), *Glycyrrhizae Radix et Rhizoma* (Gan Cao).	heat-clearing and detoxifying	7d (250 mL, twice daily)	diarrhea↓, tenesmus↓,5-HT↓, ZO-1 ↑	RCT^1^, single-blind	Damp-Heat Syndrome	colon or rectal cancer	regulating the composition of gut microbiota, enhancing intestinal barrier function
Shen (2021) (93)	119	Oral	*Qing-Chang-Hua-Shi granules:* *Coptidis Rhizoma* (Huang Lian)*, Scutellariae Radix* (Huang Qin)*, Rhizoma Sinensis* (Bai Jiang Cao)*, Radix Angelicae Sinensis* (Dang Gui)*, Radix Paeoniae Albae* (Bai Shao)*, Radix Sanguisorbae (Di Yu), Radix Arnebiae* (Zi Cao)*, Radix et Rhizoma Rubiae* (Qian Cao)*, Radix Angelicae Dahuricae* (Bai Zhi)*, Radix Aucklandiae (Mu Xiang), Radix et Rhizoma Glycyrrhizae* (Gan Cao).	heat-clearing and detoxifying	125g, twice daily	mucous and bloody stools↓, Mayo score↓, mucosal response rate, and mucosal healing rate↑	RCT, Multic-enter, double-blind	–	UC^2^	preventing inflammatory responses, inhibiting apoptosis through the MEK/ERK signaling pathway and protecting intestinal epithelial cells
Xu (2019) (94)	124	Enema	*Baitouweng Decoction*: *Pulsatillae Radix* (Bai Tou Weng), *Phellodendri Cortex* (Huang Bai), *Coptidis Rhizoma* (Huang Lian), and *Fraxini Cortex* (Qin Pi).	heat-clearing and detoxifying, Eliminate Dampness	once daily, for 30days	Improving symptoms and shortening hospitalization time.	RCT	Damp-Heat Syndrome	UC	improve intestinal function, and promote the restoration of cellular immune function.
Wu (2024) (95)	86	Oral	*Qingre Zaoshi Jiedu Huyin Decoction*: *Notoginseng Radix et Rhizoma* (San Qi, fried), *Atractylodis Macrocephalae Rhizoma* (Bai Zhu, fried), *Poria* (Fu Ling), *Dioscoreae Rhizoma* (Shan Yao, fried), *Lablab Semen Album* (Bai Bian Dou), *Coicis Semen* (Yi Yi Ren), *Platycodonis Radix* (Jie Geng), *Amomi Fructus* (Sha Ren, taken at the end), *Daemonoropis Resina* (Xue Jie), *Coptidis Rhizoma* (Huang Lian), *Magnoliae Officinalis Cortex* (Hou Po), *Pulsatillae Radix* (Bai Tou Weng), *Atractylodis Rhizoma* (Cang Zhu), *Citri Reticulatae Pericarpium* (Chen Pi), *Paeoniae Radix Alba* (Bai Shao), and *Glycyrrhizae Radix et Rhizoma* (Gan Cao).	Clear heat, dry dampness, detoxify, and protect yin.	120 to 240 mL, once daily, for 2 months.	Improvement in symptoms such as urgency with a feeling of incomplete bowel evacuation, diarrhea, abdominal pain, bloating, blood in stools, loss of appetite, and fatigue.	RCT	Damp-heat syndrome, spleen yin deficiency.	UC	–
Peng (2024) (96)	80	bamboo scraping therapy combined with acupoint application	*Shaoyao Decoction* for acupuncture point application: *Paeoniae Radix Alba* (Bai Shao), *Arecae Semen (*Bing Lang), *Angelicae Sinensis Radix* (Dang Gui), *Rhei Radix et Rhizoma* (Da Huang), *Coptidis Rhizoma* (Huang Lian), *Scutellariae Radix* (Huang Qin), *Aucklandiae Radix* (Mu Xiang), *Cinnamomi Cortex* (Rou Gui), and *Glycyrrhizae Radix et Rhizoma* (Gan Cao).	“TongYinTongYong”(Treating the root cause with universal applicability)	bamboo scraping twice a week in a cycle, acupoint application once daily for 2 weeks.	Improvement in symptoms such as diarrhea, abdominal bloating, abdominal pain, purulent blood stools, and tenesmus.	RCT	Damp-Heat Syndrome	UC	Protecting the patient’s intestinal mucosa, reducing inflammation, and regulating immune function.
Peng (2022) (97)	1	Oral	*Jianpi Qingre Huashi Granular Decoction: Codonopsis Radix (Dang Shen), Atractylodis Macrocephalae Rhizoma (Bai Zhu), Poria (Fu Ling), Aucklandiae Radix (Mu Xiang), Agastachis Herba (Huo Xiang), Puerariae Lobatae Radix (Ge Gen), Coptidis Rhizoma (Huang Lian), Dioscoreae Rhizoma (Shan Yao), Hordei Fructus Germinatus (Mai Ya), Notoginseng Radix et Rhizoma (San Qi), Atractylodis Rhizoma (Cang Zhu), Coicis Semen (Yi Yi Ren), and Sanguisorbae Radix Carbonisata (Di Yu Tan).*	Strengthen the spleen, clear heat and transform dampness, regulate qi and relieve pain, activate blood circulation, and stop bleeding	150ml, once daily, for 30days	Improvement in the scores of diarrhea, bloating, physical fatigue, and the total TCMSS score.	N-of-1 Trial	Spleen deficiency with damp-heat	UC	–
He (2024) (98)	60	Oral	*Xuchangqing* (Xuchangqing), *Coptidis Rhizoma* (Huang Lian), *Scutellariae Radix* (Huang Qin), *Aucklandiae Radix* (Mu Xiang, processed), *Paeoniae Lactiflorae Radix* (Shao Yao, fried), and *Glycyrrhizae Radix et Rhizoma* (Gan Cao), among others.	Clear Heat, Eliminate Dampness, Promote Qi Circulation, and Relieve Pain	twice daily for 8weeks	Improvement in symptoms such as diarrhea, bloody mucous stools, abdominal pain, bloating, anal burning, and tenesmus.	RCT	Internal Damp-Heat Syndrome	UC	–
Ben-Horin (2024) (99)	42	Oral	Curcumin-QingDai Combination	–	3 capsules daily for 4 weeks	Increase in clinical remission rate and improvement in endoscopy, CYP1A1 ↑	double-blind, randomized, placebo-controlled	–	ulcerative colitis	Induction of the transcription factor AhR translocation from the cytoplasm to the nucleus, thereby inducing the expression of the CYP1A1 and CYP1A2 genes.
Erol Doğan (2024) (100)	48	Oral	Curcumin, and Resveratrol	–	two capsules daily,8 weeks	Disease activity↓, inflammation↓, quality of life↑	prospective multicenter three-arm RCT	–	ulcerative colitis	–
Guo (2022) (101)	63	Acupuncture and Moxibustion	–	mechanical or thermal stimulation	12-week	CDAI↓,TGF-β 1↓,TβR2↓,Smad3↓, Snail↓	RCT, single-blind	–	Crohn’s Disease	transforming growth factor β 1 (TGF- β 1)/Smad3/Snail pathway.
Qi (2021) (102)	69	moxibustion	Qihai (CV6),bilateral Tianshu (ST25),Shangjuxu (ST37)	stimulate channel-qi	randomized, single-blind	SDS↓, SAS↓, IBDQ↑	RCT, single-blind	–	ulcerative colitis	toll-like receptors 4 signaling pathways
Guo (2024) (103)	60	Oral	Huanglian Wendan Tang (Coptis and Warm Gallbladder Decoction): *Pinelliae Rhizoma Praeparatum* (Fa Ban Xia), *Aurantii Fructus Immaturus* (Zhi Shi), *Bambusae Caulis in Taeniam* (Zhu Ru), *Citri Reticulatae Pericarpium* (Chen Pi), *Poria* (Fu Ling), *Coptidis Rhizoma* (Huang Lian), *Glycyrrhizae Radix et Rhizoma* (Gan Cao), *Zingiberis Rhizoma Recens* (Sheng Jiang).	Clear the intestines and resolve dampness, purge heat and relieve pain, tonify the spleen and kidney.	twice daily for 4weeks	The time to clinical symptom relief↓, Mayo score↓, UCEIS score.↓	RCT	Internal Damp-Heat Syndrome	ulcerative colitis	TNF-α↓, NF-κB↓, TLR4↓, IL-10↑
Fan (2020) (104)	60	Oral	Anchang Yuyang Decoction combined with mesalazine:*Astragali Radix* (Sheng Huang Qi), *Atractylodis Macrocephalae Rhizoma Praeparatum* (Chao Bai Zhu), *Coicis Semen* (Yi Yi Ren), *Patriniae Herba* (Bai Jiang Cao), *Coptidis Rhizoma*(Huang Lian), *Scutellariae Radix* (Huang Qin), *Aucklandiae Radix* (Mu Xiang), *Arecae Semen* (Bing Lang), *Sanguisorbae* Radix Carbonized (Di Yu Tan), *Bletillae Rhizoma* (Bai Ji), *Angelicae Sinensis Radix* (Dang Gui), *Paeoniae Radix Alba Praeparata* (Chao Bai Shao), *Saposhnikoviae Radix* (Fang Feng), *Glycyrrhizae Radix et Rhizoma* (Sheng Gan Cao).	Treat both the root cause and symptoms, support the righteous qi and expel pathogens, and combine attacking and tonifying strategies.	once daily,for 12-week	Lesion reduction and symptom relief.	RCT	spleen deficiency and dampness retention	ulcerative colitis	TNF-α↓, IL-10↑
Dai (2024) (105)	140	anus	Qingchang suppositry:Indigo Naturalis (Qing Dai), Portulacae Herba (Ma Chi Xian), Notoginseng Radix et Rhizoma (San Qi), Galla Chinensis (Wu Bei Zi), Borneolum Syntheticum (Bing Pian)	clear heatness and eliminate dampness, improve blood circulation and disperse stasis, stop bleeding and promote ulcer healing.	twice daily for 12weeks	relieve the symptoms, improve mucosa healing and ameliorate histological inflammation	RCT	Damp-Heat Syndrome	ulcerative colitis	–
Zhou (2021) (106)	14	Enema	Baitouweng Tang.(Pulsatilla Decoction): *Pulsatillae Radix* (Bai Tou Weng), *Phellodendri Chinensis Cortex* (Huang Bo), *Fraxini Cortex* (Qin Pi), *Coptidis Rhizoma* (Huang Lian), *Bletillae Rhizoma* (Bai Ji), *Sanguisorbae Radix* (Di Yu), *Notoginseng Radix et Rhizoma* (San Qi), *Aucklandiae Radix* (Mu Xiang), *Citri Reticulatae Pericarpium* (Chen Pi).	Clear heat and detoxify, cool the blood and relieve dysentery.	once daily,for 6weeks	Improve intestinal mucosal injury and enhance endoscopic scores.	RCT	Damp-Heat Syndrome	ulcerative colitis	–

^1^ RCT, Randomized controlled trial.

^2^ UC, ulcerative colitis. "↑" means an increase, while "↓" represents a decrease in expression.

Fifty articles focused on treatments such as oral Chinese medicine, bamboo scraping therapy, acupoint application, acupuncture, and moxibustion. Due to the limited number of irColitis cases, conducting large-scale clinical trials remains challenging. Therefore, as indicated in **Table 4**, most of the reviewed studies explore herbal formulas for the treatment of ulcerative colitis and Crohn’s disease. However, a study has explored the use of Chinese patent medicine (105). Given its convenience and cost-effectiveness, further research in this area is warranted.

In TCM, acute inflammatory symptoms are typically associated with Damp-Heat Syndrome. Treatment focuses on clearing heat, detoxifying, and drying dampness, using classic formulas such as Gegen Qinlian decoction, Baitouweng decoction, and Shao Yao decoction. Frequently used herbs include *Phellodendri Cortex* (Huang Bai), *Coptidis Rhizoma* (Huang Lian), *Scutellariae Radix* (Huang Qin), and *Rhei Radix et Rhizoma* (Da Huang) for their heat-clearing, damp-drying, and detoxifying properties. In TCM theory, it is commonly believed that the patient enters a stage of “deficiency” (Zheng Xu), then treatment focused on tonifying the spleen and stomach for improving patient’s condition. Additionally, TCM emphasizes supporting the body’s vital energy (Zheng Qi) with herbs such as *Atractylodis Macrocephalae Rhizoma* (Bai Zhu), *Glycyrrhizae Radix et Rhizoma* (Gan Cao), and *Dioscoreae Rhizoma* (Shan Yao).

## 6 Future research directions and challenges

The widespread use of ICIs in oncology has led to increasing clinical and research interest in irColitis. Although increasing findings on irColitis published (8), studies on epidemiological characteristics and underlying mechanisms remain insufficient, needing future researches.

### 6.1 In-depth mechanistic studies

The exploration of mechanisms on the effect Chinese medicine and integrative therapies for irColitis remains limited. Some studies demonstrated the potential interactions between TCM and ICIs, as well as the underlying mechanisms of integrated TCM and Western medicine in the treatment of irColitis. A meta-analysis evaluating the efficacy, safety, and potential mechanisms of TCM as an adjuvant therapy in cancer immunotherapy indicated that TCM influenced PD-1/PD-L1 inhibitors through tumor microenvironment modulation, gut microbiota regulation, inhibition of PD-1 or PD-L1 expression, and cytokine signaling regulation (107). Previous studies on inflammatory bowel diseases IBD with similar symptoms, such as Crohn’s disease and ulcerative colitis, suggested that Chinese medicine might alleviate irColitis through mechanisms including immune regulation, reduction of intestinal inflammation, modulation of gut microbiota, and protection of the intestinal mucosal barrier (2). This highlights the great potential of integrative traditional Chinese and Western medicine in treatment.

For example, the JAK/STAT and NF-κB signaling pathways may serve as important potential targets. PD-1/PD-L1 expression is regulated by JAK/STAT and NF-κB signaling pathways, both of which are targeted by TCM like Baicalin and Gegen Qinlian decoction (108–110). Experimental studies have demonstrated that Baicalin is an effective treatment for IBD. By restoring the Th17/Treg balance through the JAK/STAT signaling pathway and reducing ZO-1 secretion, Baicalin alleviates intestinal inflammation and preserves the integrity of the intestinal epithelial barrier, thereby improving clinical symptoms (108). This signaling pathway is particularly important during the acute phase of irColitis. On the other hand, research suggested that the inhibition of Toll-like receptor 4/Nuclear factor kappa B (TLR4/NF-κB) signaling and the enhancement of gut microbiota abundance might be key mechanisms the therapeutic efficacy of Gegen Qinlian decoction as well as other TCM decoctions in the treatment of IBD (109, 110). Additionally, traditional herbal medicines, such as Sophora flavescens Aiton, P. grandiflorus, and Kuijieling decoction, have also shown significant effects in restoring gut immune function. These herbs achieve this by suppressing excessive T cell activation and rebalancing Th17/Treg (108, 111, 112). Research on P. grandiflorus in the treatment of IBD has revealed that this herbal remedy modulates the homeostasis of colonic immune cells through the mesenteric lymphatic circulation, offering valuable insights for future research directions (113).

Meanwhile, TCM may exert its effects through different targets depending on the progression of irColitis. In the acute phase of irColitis, characterized by severe inflammation and epithelial barrier disruption, TCM interventions targeting rapid immune modulation and inflammation control may be particularly beneficial. For example, Baicalin and Gegen Qinlian decoction have been shown to effectively reduce severe acute bowel inflammation through JAK/STAT and TLR4/NF-κB signaling passageway, regulating the secretion of pro-inflammatory cytokine such as IL-17, IL-6, and IL-1β (108–110). When combined with corticosteroids, they may help achieve faster symptom control. Additionally, the multi-targeted mechanisms of TCM can alleviate the side effects associated with corticosteroid use, thereby improving patient tolerance and adherence to treatment.

The gut microbiome plays a critical role in the pathogenesis of irColitis and represents a promising area for further research. In chronic irColitis, where immune dysregulation and microbiota imbalance persist, TCM’s long-term regulatory effects on gut microbiota and immune homeostasis may be advantageous. Existing studies have demonstrated that various herbal medicines possess the ability to improve gut microbiota abundance (2). While current studies suggested that gut microbiota significantly influences the development of irColitis (3, 71, 77), future investigations should focus on elucidating the complex interactions between ICIs, gut immune function, and the microbiome. A deeper understanding of these interactions is essential for advancing prevention and treatment strategies and clarifying the mechanisms by which Chinese medicine affects chronic irColitis.

### 6.2 Treatment strategy optimization—the promising potential of TCM

Currently, the treatment of irColitis primarily relies on corticosteroids and immunosuppressants such as Infliximab and Vedolizumab. However, some patients show inadequate responses to conventional treatments, and there is even the emergence of drug resistance. Therefore, future research should focus on exploring new therapeutic strategies.

In the treatment of irColitis, TCM emphasizes restoring balance within the body, particularly by normalizing the immune system and gut function. TCM theories, including those related to the gut microbiome, spleen and stomach function, and damp-heat, offer valuable insights for treating irColitis. This approach holds significant potential for future therapeutic strategies. Moreover, TCM emphasizes individualized treatment, tailoring therapeutic strategy according to each patient’s unique symptoms and physical constitution. In the future, combining individualized TCM approaches could enhance the therapeutic efficacy of irColitis.

The integration of TCM and Western medicine holds significant potential for the treatment of irColitis. During the acute phase, irColitis is often characterized by symptoms such as diarrhea, mucus or bloody stools, and abdominal pain. In severe cases, patients may experience serious complications such as intestinal perforation and sepsis, necessitating rapid inflammation control. Western medicine primarily focuses on immediate immunosuppression through glucocorticoids and biologics. Concurrently, anti-inflammatory Chinese herbal medicines, such as *Scutellariae Radix* (Huang Qin), *Coptidis Rhizoma* (Huang Lian), and *Rhizoma Sinensis* (Bai Jiang Cao), may be used in combination (93). Additionally, spleen-tonifying herbs like *Codonopsis Radix (Dang Shen)*and *Atractylodis Macrocephalae Rhizoma* (Bai Zhu) may help protect the intestinal barrier (97). Future research should further explore the role of integrative therapy in reducing glucocorticoid dosage, shortening treatment duration, and mitigating drug resistance during the acute phase. In the chronic phase or in cases of prolonged disease due to repeated ICI treatment, Western medicine offers limited strategies. At this stage, TCM presents a promising approach, interpreting the condition as an underlying deficiency with excess manifestations (Ben Xu Biao Shi). TCM emphasizes strengthening and protecting the spleen and stomach, often employing formulas that tonify the spleen and dry dampness, with individualized modifications. Studies have shown that Shenling Baizhu San improves colitis by enhancing intestinal epithelial barrier integrity and reducing inflammation (114). Additionally, a meta-analysis suggested that it alleviates diarrhea symptoms by enhancing immune function (115). Therefore, integrating Chinese medicine into standard Western treatment, tailored to the patient’s specific symptoms at different stages of irColitis represents a highly promising therapeutic approach. Previous studies on ulcerative colitis have demonstrated that integrative treatment combining TCM and Western medicine yields superior efficacy compared to either treatment alone (104, 106).

However, clinical evidence supporting the use of TCM or integrative approaches for irColitis remains limited. Current clinical practices mainly follow the “treating different diseases with similar symptoms (Yi Bing Tong Zhi)” principle, often based on treatment strategies for diseases with analogous symptoms. Therefore, further high-quality clinical trials are required. Future research should focus on evaluating the clinical efficacy of TCM in treating irColitis, assessing its safety, and exploring its synergistic effects with corticosteroids and immunosuppressants. Multicenter, large-scale, randomized controlled, double-blind trials will be essential to verify the therapeutic effects of TCM in irColitis and provide scientific evidence for its broader clinical application.
