# Supplementary material for: An updated review on immune checkpoint inhibitor-induced colitis: epidemiology, pathogenesis, treatment strategies, and the role of traditional Chinese medicine
Source: Front Immunol. 2025 Mar 17;16:1551445. doi: 10.3389/fimmu.2025.1551445 (PMC11955479; doi:10.3389/fimmu.2025.1551445)
Supplement: Supplementary file 1 [file SupplementaryFile1.docx]

**supplementary table 1. data on dosage and duration**

| **Author**  **(Year)** | **Drugs and Dosages** | **Duration(Immunotherapy end points)** |
| --- | --- | --- |
| Diaz(10)  (2022) | pembrolizumab 200 mg q3w | Treatment continued until disease progression, unacceptable toxicity, illness, patient/physician decision to withdraw, or completion of a maximum of 35 pembrolizumab treatments. |
| Shitara(11)  (2024) | pembrolizumab 200 mg q3w combined with cisplatin-based chemotherapy | Pembrolizumab combined with cisplatin-based chemotherapy for 3 cycles. This was followed by 11 cycles of adjuvant pembrolizumab. |
| Lynch(12)  (2023) | A therapy cycle was administered every 28 days, with doses of AVD (doxorubicin [IV, 25 mg/m²], vinblastine [IV, 6 mg/m²], and dacarbazine [IV, 375 mg/m²]) on days 1 and 15, according to the standard dosing regimen.pembrolizumab 200 mg q3w | All patients were required to receive at least 2 cycles of the experimental treatment, with a maximum of 6 cycles allowed, as determined by the investigator based on staging and baseline risk factors. |
| Ready(13)  (2023) | nivolumab (240 mg q2w) +ipilimumab (1 mg/kg q6w) | Until disease progression, unacceptable toxicity, withdrawal of consent, or for up to 2 years |
| Tykodi(14)  (2022) | nivolumab(3 mg/kg)+ ipilimumab(1 mg/kg) | Nivolumab 3 mg/kg plus ipilimumab 1 mg/kg every 3 weeks for up to four doses followed by nivolumab 480 mg every 4 weeks for up to 2 years or until disease progression, unacceptable toxicity, withdrawal of consent, or end of trial, whichever occurred first. |
| Frentzas(15)  ( 2024) | Ivonescimab(d1 and d15 of q28d) 0.3、1、3、10、20 and 30 mg/kg | Ivonescimab was administered intravenously on day 1 and day 15 of each 28-day treatment cycle until disease progression, consent withdrawal or intolerable toxicity occurred. |
| Schoenfeld(16)  (2022) | durvalumab 1,500 mg q4w/tremelimumab 75 mg q4w+ HFRT5 | Durvalumab for a maximum of 13 cycles, Tremelimumab for a maximum of four cycles. |
| Oaknin(17)  (2024) | [nivolumab 240 mg q2w](https://www.sciencedirect.com/topics/pharmacology-toxicology-and-pharmaceutical-science/nivolumab) | Treatment continued until disease progression, unacceptable toxicity, or consent withdrawal, or for up to 24 months. |
|  | nivolumab 3 mg/kg q2w + ipilimumab 1 mg/kg q6w |  |
|  | nivolumab 1 mg/kg q3w+ ipilimumab 3mg/kg q3w for 4cycles,followed by nivolumab 240 mg q2w |  |
| Monge(18)  (2023) | PexaVec+durvalumab 1500 mg q28d | Treatment continued until disease progression, unacceptable toxicity or withdrawal of consent. |
|  | PexaVec+a single dose of tremelimumab(day1)+durvalumab 1500 mg q28d |  |
| Necchi(19)  (2024) | pembrolizumab 200 mg q3w | Patients received pembrolizumab 200 mg intravenously every 3 weeks for a maximum of 35 cycles. |
| Grimm(20)  (2023) | nivolumab 240 mg q2w;nivolumab 3mg/kg+ipilimumab 1 mg/kg q3w | The primary endpoint was confirmed investigator-assessed objective response rate in the full analysis set |
| Lakhani(21)  (2024) | retifanlimab 3 mg/kg q2w | Treatment with retifanlimab continued for ≤2 years or until disease progression, alternative cancer therapy initiation, unacceptable toxicity, withdrawal of consent, or other reason for drug discontinuation. |
| Schöffski(22)  ( 2023) | olaratumab(15or20 mg/kg,d1d8)+ pembrolizumab(200 mg,d1) |  |
| Zhao(23)  (2024) | SBRT(24 Gy in three fractions)with sequential tislelizumab(2 cycles of 200 mg) and chemotherapy | Surgical resection was performed 4–6 weeks after neoadjuvant treatment. |
| Saba(24)  (2024) | nivolumab 240mg q2w and IMRT^6^ reirradiation | 52 weeks. |
| Emamekhoo(25)  (2022) | nivolumab 3mg/kg +ipilimumab 1mg/kg q3w;followed by nivolumab 480mg/4weeks | Patients in cohort 3 received NIVO 3 mg/kg plus IPI 1 mg/kg every 3 weeks for up to 4 doses intravenously followed by NIVO 480 mg every 4 weeks until disease progression, unacceptable toxicity, withdrawal of consent, or the end of the trial, whichever occurred first, or up to a maximum of 2 years. |
| Morano(26)(2022) | temozolomide+nivolumab 480 q4w+ipilimumab 1 mg/kg q8w | Patients received the study treatments until RECIST1.1 PD, unacceptable toxicity, consent withdrawal, death, or immune-related RECIST (ir-RECIST) PD |
| Kim(27)  (2024) | atezolizumab 800 mg,q2w+Chemotherapy | Up to 1 year |
| George(7)  (2022) | nivolumab 6 mg/kg+ipilimumab 1 mg/kg q8w; alternating with nivolumab 480 mg q8w,  staggered q4w | Treatment continued until disease progression, unacceptable toxicity, withdrawal of consent or end of study. The maximum treatment duration was 2 years. |
| Ferris(28)  (2022) | Cetuximab, Radiotherapy, and Ipilimumab(1,3,10mg/kg) |  |
| Xiao(29)  (2022) | Pembrolizumab 200 mg q3w and SBRT^7^ |  |
| Marabelle(30)  (2020) | pembrolizumab200mg q3w | 35 cycles—approximately 2 years—or until documented disease progression, unacceptable toxicity, intercurrent illness preventing additional treatment administration, or patient/investigator decision. |
| Stratigos(31)  (2021) | cemiplimab 350 mg q3w | Up to 93 weeks or until progression or unacceptable toxicity. |
| Qian(32)  (2021) | ipilimumab, nivolumab, or pembrolizumab, or a combination of these |  |
| Tawbi(33)  (2021) | nivolumab 1 mg/kg +ipilimumab 3 mg/kg q3w; nivolumab 3 mg/kg | Patients received nivolumab 1 mg/kg combined with ipilimumab 3 mg/kg both intravenously once every 3 weeks for 12 weeks (for a total of four doses; induction phase), followed by nivolumab 3 mg/kg intravenously every 2 weeks for a total of 24 months or until progression or unacceptable toxicity (maintenance phase). |
| Ascierto(34)  (2020) | ipilimumab 10 mg/kg q3w | Nivolumab 3 mg/kg every 2 weeks for four doses, and then every 12 weeks until 1 year of treatment, disease recurrence, unacceptable toxicity, or withdrawal of consent. |
| Goldberg(35)  (2020) | pembrolizumab 10mg/kg q2w | Pembrolizumab 10mg/kg IV every two weeks for up to 24 months or until disease progression (without clinical benefit) or unacceptable toxicity. |
| Campbell(36)  (2021) | tremelimumab 10mg/kg q4w | The dose of tremelimumab was 10 mg/kg intravenous infusion every 4 weeks for two doses followed by tissue collection with the resumption of tremelimumab every 4 weeks for three additional doses followed by every 12-week administration. |
| Cacciotti(37)  (2020) | ipilimumab,nivolumab and/or pembrolizumab |  |
| Gao(38)  (2020) | durvalumab (1500 mg)+tremelimumab (75 mg) | Two cycles of durvalumab (1500 mg) plus tremelimumab (75 mg) on weeks 1 and 5 |
| Brastianos(39)  (2021) | nivolumab+ipilimumab, dosage vary depending on the type of cancer. | The dosing regimens specific to primary tumor histology were administered intravenously as directed by the manufacturer’s (Bristol Meyers Squibb) guidelines,The primary endpoint was the rate of overall survival at 3 months. |
| Kawazoe(40)  (2020) | Pembrolizumab 200 mg q3w | SOX with pembrolizumab treatment was continued until progressive disease (PD), unacceptable toxicity or withdrawal of consent occurred or until patients had received 35 cycles of pembrolizumab. |
| Tolaney(41)  (2020) | pembrolizumab, 200 mg, q3w |  |
| Sanborn(42)  (2021) | pacmilimab (0.3, 1, 3, or 10 mg/kg) +ipilimumab (3 or 6 mg/kg) q3w for 4 doses, followed by pacmilimab monotherapyq2w. | Permanent discontinuation was also required or if grade 2 or 3 treatment-related AEs did not improve to grade ≤1 or resolve within 12 weeks of the most recent dose of pacmilimab. All patients continued treatment until disease progression, unacceptable toxicity, or withdrawal of consent. |
| Desai(43)  (2020) | tislelizumab |  |
| Diefenbach(44)  (2020) | brentuximab1-8mg/kg, nivolumab3mg/kg, and ipilimumab1mg/kg | The maximum duration of brentuximab was 1 year (16 doses), and for nivolumab 2 years (34 doses), ipilimumab 2 years (9 doses). Patients were removed from study due to patient preference, progression of disease, toxicity, or non-compliance. |
| Apolo(45)  (2020) | cabozantinib 40 mg/d, nivolumab 3 mg/kg, and ipilimumab 1 mg/kg | Patients could discontinue treatment as a result of PD, unacceptable toxicity, or withdrawal of consent or based on the investigator’s clinical judgment. |
| Yap(46)  (2021) | pembrolizumab 200 mg q3w | Up to 35 cycles |
| McDermott(47)  (2021) | pembrolizumab 200 mg q3w | Study treatment was continued until confirmed progressive disease (PD); unacceptable toxicity or intercurrent illness that prevented further administration of treatment; 35 doses of pembrolizumab had been received; or withdrawal of consent, whichever occurred first. |
| Boutros(48)  (2020) | ipilimumab at 10 mg/kg q3w | Patients without progressive disease (PD) who tolerated the treatment continued ipilimumab dosing in 12-week intervals until progression or withdrawal of consent. |
| Felip(49)  (2020) | nivolumab 3mg/kg q2w | Until disease progression, unacceptable toxicity or withdrawal of consent |

| **Abbreviation** | **Full Name** | **Explanation** |
| --- | --- | --- |
| ICIs | Immune checkpoint inhibitors | Cancer immunotherapy drugs that block inhibitory signals on T cells. |
| SCFAs | Short-chain fatty acids | Metabolic products of gut microbiota with immunomodulatory effects. |
| IFN-γ | Interferon-gamma | A cytokine that activates macrophages and promotes Th1 responses. |
| TNF-α | Tumor necrosis factor-alpha | A pro-inflammatory cytokine involved in immune activation. |
| IL-6 | Interleukin-6 | A cytokine that regulates inflammation and immune responses. |
| IL-1β | Interleukin-1 beta | A cytokine that promotes inflammation and fever response. |
| ZO-1 | Zonula occludens-1 | A tight junction protein essential for epithelial barrier integrity. |
| Th1 | T-helper 1 cells | A subset of T cells that produce IFN-γ and mediate cellular immunity. |
| Th17 | T-helper 17 cells | A subset of T cells that produce IL-17 and drive inflammation. |
| NLRP3 | Nucleotide-binding oligomerization domain-like receptor pyrin domain-containing 3 | A key inflammasome component involved in IL-1β activation. |
| ITGB2 | Integrin beta-2 | A protein involved in immune cell adhesion and signaling. |
| ICAM | Intercellular adhesion molecule | A molecule that facilitates immune cell interactions. |

**Supplementary Table 2.** Abbreviations and Explanations

| irColitis | Immune checkpoint inhibitor-induced colitis | Colitis induced as an adverse reaction to immune checkpoint inhibitors. |
| --- | --- | --- |
| irAEs | Immune-related adverse events | Adverse events related to immune system activation during treatment with immunotherapies. |
| TCM | Traditional Chinese Medicine | A holistic medical system originating in China, utilizing herbs, acupuncture, and other therapies. |
| RA | Rheumatoid arthritis | An autoimmune disease characterized by inflammation in joints. |
| NSCLC | Non-small cell lung cancer | A type of lung cancer that is not classified as small cell lung cancer. |
| RCC | Renal cell carcinoma | A type of kidney cancer originating from the renal tubules. |
| CRC | Colorectal cancer | Cancer affecting the colon or rectum. |
| HNSCC | Head and neck squamous cell carcinoma | A cancer affecting the mucous membranes of the head and neck. |
| IBD | Inflammatory bowel disease | Chronic inflammation of the digestive tract, including Crohn's disease and ulcerative colitis. |
| CTLA-4 | Cytotoxic T-lymphocyte-associated antigen 4 | A protein receptor on T cells that downregulates immune responses. |
| PD1 | Programmed cell death protein 1 | A protein receptor on T cells that inhibits immune responses when bound to its ligands. |
| TRMs | Tissue-resident memory T cells | Specialized T cells that remain in tissues after infection, providing rapid immune responses. |
